# Risk factors for difficult endoscopic hemostasis for colonic diverticular bleeding and efficacy and safety of transcatheter arterial embolization

**DOI:** 10.1097/MD.0000000000035092

**Published:** 2023-09-15

**Authors:** Tomoe Sano, Toru Ishikawa, Motoi Azumi, Ryo Sato, Ryo Jimbo, Yuji Kobayashi, Toshifumi Sato, Akito Iwanaga, Junji Yokoyama, Terasu Honma

**Affiliations:** a Department of Gastroenterology and Hepatology, Saiseikai Niigata Hospital, Niigata, Japan.

**Keywords:** colonic diverticular bleeding, endoscopic hemostasis, recurrence-free survival, risk factors, transcatheter arterial embolization

## Abstract

This study aimed to investigate the risk factors for difficult endoscopic hemostasis in patients with colonic diverticular bleeding and to evaluate the efficacy and safety of transcatheter arterial embolization (TAE) for colonic diverticular bleeding. This study included 208 patients with colorectal diverticular hemorrhage. The non-interventional radiotherapy group consisted of patients who underwent successful spontaneous hemostasis (n = 131) or endoscopic hemostasis (n = 56), whereas the interventional radiotherapy group consisted of patients who underwent TAE (n = 21). Patient clinical characteristics were compared to identify independent risk factors for the interventional radiotherapy group. Furthermore, the hemostasis success rate, rebleeding rate, complications, and recurrence-free survival were compared between patients who underwent endoscopic hemostasis and those who underwent TAE. Bleeding from the right colon (odds ratio [OR]: 7.86; 95% confidence interval [CI]: 1.6–38.8; *P* = .0113) and systolic blood pressure <80 mm Hg (OR: 0.108; 95% CI: 0.0189–0.62; *P* = .0126) were identified as independent risk factors for the interventional radiology group. The hemostasis success rate (*P* = 1.00), early rebleeding rate (within 30 days) (*P* = .736), late rebleeding rate (*P* = 1.00), and recurrence-free survival rate (*P* = .717) were not significantly different between the patients who underwent TAE and those who underwent endoscopic hemostasis. Patients in the TAE group experienced more complications than those in the endoscopic hemostasis group (*P* < .001). Complications included mild intestinal ischemia (19.0%) and perforation requiring surgery (4.8%). Patients who required interventional radiotherapy were more likely to bleed from the right colon and presented with a systolic blood pressure of <80 mm Hg. TAE is an effective treatment for patients with colonic diverticular hemorrhage that is refractory to endoscopic hemostasis. However, complications must be monitored carefully.

## 1. Introduction

The number of patients with colorectal diverticula is increasing in Japan owing to the aging population, as well as the Westernized diet and habits.^[1]^ Furthermore, the use of antithrombotic agents has increased the number of patients with diverticular hemorrhage.^[2,3]^ Diverticular hemorrhage accounts for 26.4% to 33% of acute lower gastrointestinal bleeding.^[4–6]^ Spontaneous hemostasis occurs in 70% to 90% of patients with diverticular hemorrhage,^[7,8]^ and some patients require hemostasis for persistent bleeding.^[9,10]^

Colonic diverticular bleeding is often diagnosed by colonoscopy. When stigmata of recent hemorrhage (SRH) are observed on colonoscopy, the risk of bleeding is high and endoscopic treatment is required.^[11–13]^ Endoscopic treatment for colonic diverticular bleeding traditionally involves clipping, coagulation, and hemostasis; nevertheless, the use of endoscopic band ligation (EBL) and indwelling snares has recently been reported.^[14–18]^ However, endoscopic hemostasis is difficult to achieve in some patients, and interventional radiology (IVR) or surgery is necessary.^[13,15,19–21]^

Colorectal diverticular bleeding refractory to endoscopic hemostasis is a severe condition. However, only a few studies have investigated the risk factors for this condition.^[10,22,23]^ Transcatheter arterial embolization (TAE) has been reported to achieve a hemostasis rate of 40% to 100%, despite a high complication rate.^[13,24–28]^ Therefore, the present study aimed to investigate the risk factors for refractory colonic diverticular bleeding requiring IVR and to evaluate the efficacy and safety of TAE.

## 2. Methods

This study was conducted in accordance with the ethical principles of the 1964 Declaration of Helsinki and approved by the Standards of the Official Conduct Committee at Saiseikai Niigata Hospital (approval no. E18-15). Written informed consent was obtained from all the patients.

This study included 208 patients diagnosed with colonic diverticular hemorrhage at our hospital between January 2012 and February 2021. Colonic diverticular hemorrhage was diagnosed when the patient reported an acute onset of painless haematochezia that was paired with a colonic diverticulum with extravasation on computed tomography (CT) or a colonic diverticulum with SRH on colonoscopy.^[11,12]^ Additionally, colonic diverticular hemorrhage was diagnosed when a colonic diverticulum was present on CT or colonoscopy in the absence of extravasation and SRH after ruling out other lower gastrointestinal bleeding lesions (e.g., colorectal cancer, Behcet disease, ulcerative colitis, and ischemic enteritis). Patients with a history of colonic diverticular bleeding were also considered to have colonic diverticular bleeding after excluding those with other diseases.

Patient age, sex, comorbidities (hypertension, dyslipidemia, and diabetes mellitus), medications (non-steroidal anti-inflammatory drugs, aspirin, antiplatelet drugs, and anticoagulants), alcohol consumption, and smoking status were retrieved from medical records. The patient blood pressure, height, weight, and laboratory findings (blood count, C-reactive protein level, creatinine level, and estimated glomerular filtration rate) at presentation were also obtained from the medical records.

Contrast-enhanced CT was performed at the discretion of the attending physician, based on the patient condition and renal function. The presence of extravasation on contrast-enhanced CT was determined by a radiologist and a gastroenterologist.

Colonoscopy was performed at the discretion of the attending physician, based on the patient condition. Polyethylene glycol was used as a pretreatment for colonoscopy, except in patients with shock. In these patients, a high-pressure enema (300 mL of slightly warm water) was used as pretreatment for colonoscopy. The colonoscopes used in this study were CF-H260AI, CF-H290I, CF-HQ290I, PCF-Q260JI, PCF-Q260AZI, and PCF-290AZI (Olympus, Tokyo, Japan). Colonoscopies were performed by a gastroenterologist, who identified the presence of SRH and colonic diverticulosis.^[11]^ If SRH was detected on colonoscopy, endoscopic hemostasis was attempted via clip, coagulation, EBL, or ligation with an indwelling snare.

If hemostasis was not achieved via endoscopy, a marking clip was placed near the bleeding site to prepare for IVR. IVR was performed for cases in which endoscopic hemostasis failed to stop the bleeding or for cases in which the bleeding site could not be identified on CT or colonoscopy and repeated bleeding occurred. Patients in whom hemostasis could not be achieved via TAE underwent surgery. In this study, none of the patients underwent surgery unless TAE failed or if intestinal perforation occurred.

Patients were divided into non-IVR (spontaneous and endoscopic hemostasis) and IVR (TAE) groups, and the demographic and clinical findings of the groups were retrospectively compared. The requirements for blood transfusion during hospitalization, blood transfusion volume, and time to rebleeding were extracted from the patients’ medical records. Statistical sample size calculations were not performed for this retrospective study. Continuous variables are presented as median or mean values and were compared using Student *t* test and Mann–Whitney *U* test. Categorical data are expressed as numbers and compared using the chi-square test and Fisher exact probability test. Logistic regression analysis was performed to calculate the odds ratios (ORs) of risk factors. Multivariate analysis was conducted for risk factors that showed statistical significance in the univariate analysis. Recurrence-free survival was compared between the groups using the log-rank test and Kaplan–Meier method.

To evaluate treatment efficacy, outcomes, rebleeding rates, and complications were compared between patients who underwent endoscopic hemostasis and those who underwent TAE. The time to re-bleeding was categorized as early (within 30 days of hospital admission) or late (30 days after hospital admission). Recurrence-free survival rates were also compared between patients who underwent endoscopic hemostasis and TAE, as described above.

All statistical analyses were conducted using the EZR software (Saitama Medical Center, Jichi Medical University, Saitama, Japan). Statistical significance was set at *P* < .05.

## 3. Results

**Figure 1.**
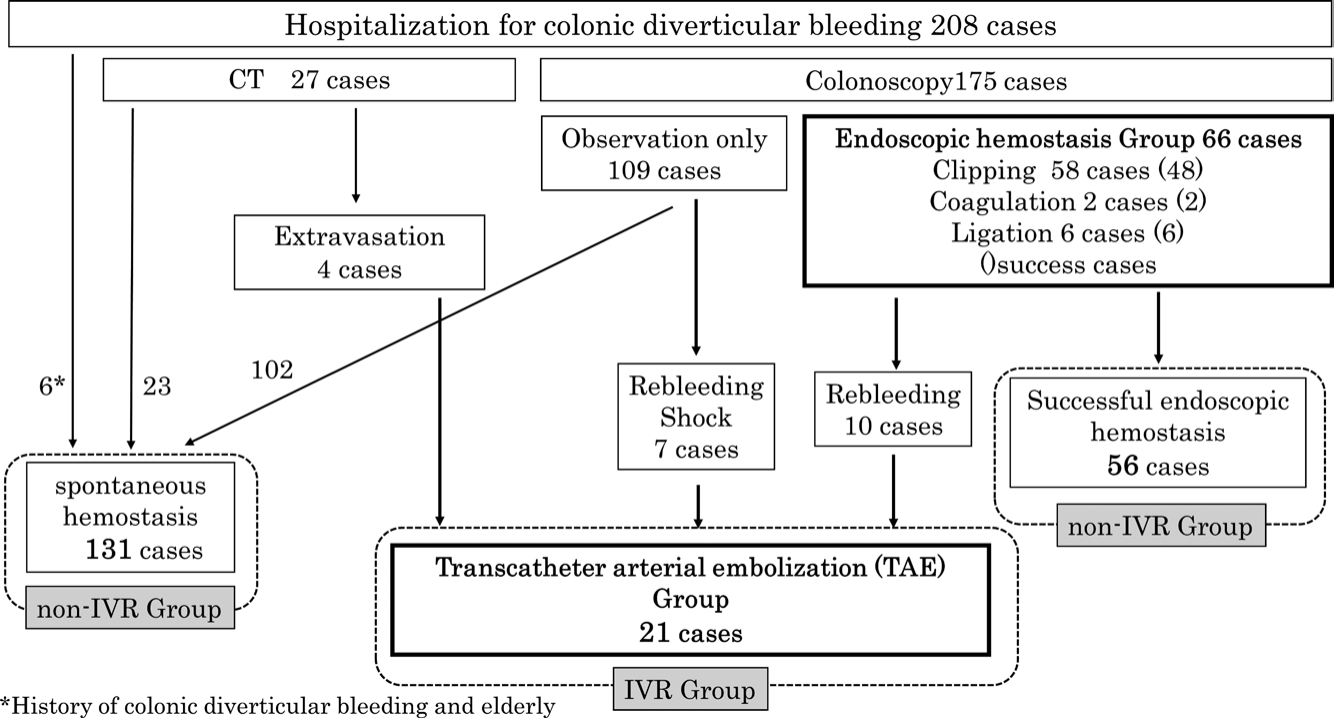
Flowchart of patients who underwent transcatheter arterial embolization (TAE) and endoscopic hemostasis for colonic diverticular bleeding. The interventional radiology group (IVR group) comprised patients who underwent transcatheter arterial embolization, whereas the non-IVR group consisted of patients who underwent successful endoscopic hemostasis and spontaneous hemostasis. IVR = interventional radiology.

Spontaneous hemostasis was achieved in 131 patients, whereas successful endoscopic hemostasis was achieved in 56 patients, resulting in 187 patients in the non-IVR group. Endoscopic treatment was performed on 66 patients. However, bleeding was not successfully stopped in 10 patients, and rebleeding or shock occurred in 7 patients because the bleeding site could not be identified on colonoscopy. Additionally, 4 patients showed clear extravasation on CT. Ultimately, 21 patients underwent TAE and were classified into the IVR group (Fig. 1).

Of the 66 patients who were treated endoscopically, 58 (87.9%) underwent endoscopic clipping, 2 (3%) underwent coagulation hemostasis, and 6 (9.1%) underwent ligation (Fig. 1). Ten patients (17.2%) who underwent clipping failed to achieve hemostasis.

**Figure 2.**
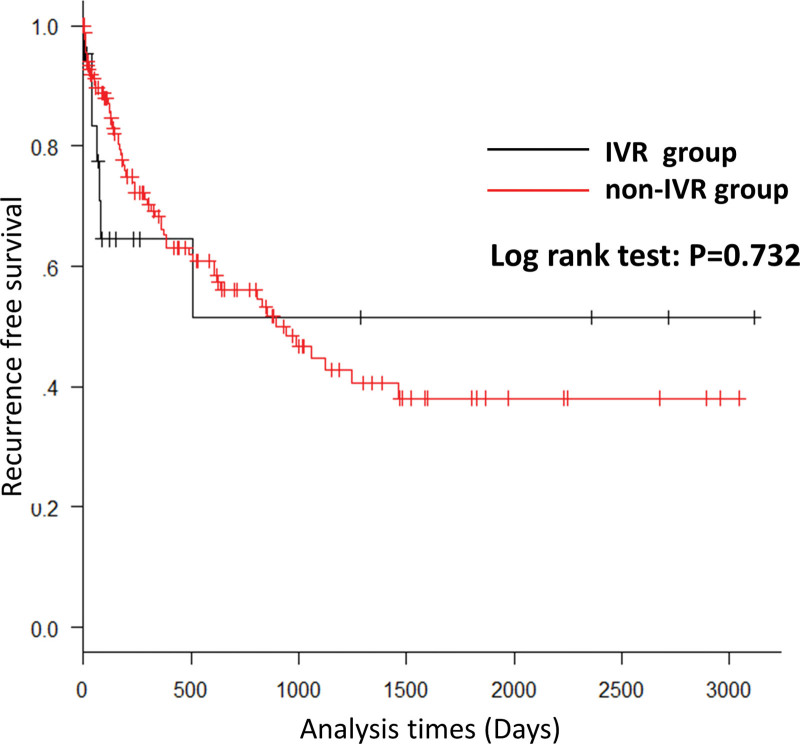
Recurrence-free survival was not significantly different between the IVR and non-IVR groups. IVR = interventional radiology.

The IVR group included more men (*P* = .005) and smokers (*P* = .011) than the non-IVR group (Table 1). The bleeding source was more commonly right-sided diverticula in patients in the IVR group (*P* < .001). More patients in the IVR group had a systolic blood pressure of < 80 mm Hg at presentation (*P* < .001), required blood transfusion (*P* < .001), and had a greater blood transfusion volume (*P* < .001). The IVR group also included taller patients (*P* = .009). CT extravasation was more commonly observed in the IVR group (*P* = .002). Bleeding from the right colon (OR: 7.86; 95% confidence interval [CI]:1.6–38.8; *P* = .0113) and systolic blood pressure < 80 mm Hg (OR: 0.108; 95% CI: 0.0189–0.62; *P* = .0126) were identified as independent risk factors for IVR (Table 1). Recurrence-free survival was not significantly different between the IVR and non-IVR groups (*P* = .732) (Fig. 2).

**Table 1:** Independent risk factors for patients requiring interventional radiology using logistic regression analysis.

	IVR group		Non-IVR group		Univariate analysis	Multivariate analysis	
			Spontaneous hemostasis (131)				
			Successful endoscopic hemostasis (56)				
	n = 21		n = 187		*P* value	OR (95% CI)	*P* value
Age (mean ± SD)	69.33 ± 15.26		74.03 ± 12.02		.1		
Male	20	95.2%	123	65.8%	**.005**	3.69 (0.17–80.6)	.407
Height (mean ± SD)	165.99 ± 8.2		158.95 ± 11.56		**.009**	1.01 (0.89–1.14)	.875
Weight (mean ± SD)	64.2 ± 8.69		60.96 ± 12.28		.253		
BMI (mean ± SD)	23.41 ± 3.15		24.02 ± 3.64		.476		
Smoking	15	71.4%	78	41.7%	**.011**	0.63 (0.15–2.71)	.537
Alcohol drinking	12	57.1%	99	52.9%	.819		
Medical history							
Colonic diverticular bleeding	11	52.4%	69	37.1%	.237		
Diabetes mellitus	7	33.3%	32	17.1%	.081		
Hypertension	13	61.9%	132	70.6%	.455		
Dyslipidaemia	4	19.0%	67	35.8%	.15		
Findings on admission							
Shock (sBP < 80 mm Hg)	8	38.1%	8	4.3%	**<.001**	**0.11 (0.02–0.62**)	**.013**
Blood transfusion (BT)	17	81.0%	65	34.8%	**<.001**	0.29 (0.07–1.2)	.089
BT (U)	8 ± 7.32		1.65 ± 2.57		**<.001**		
Laboratory data							
Hb, g/dL (mean ± SD)	9.51 ± 2.69		10.47 ± 2.5		.102		
WBC (mean ± SD)	7738.1 ± 3747.34		7137.67 ± 2568.9		.336		
CRP (mean ± SD)	0.37 ± 1.04		0.5 ± 1.15		.618		
Creatinine (mean ± SD)	1.23 ± 1.34		1.2 ± 2.45		.966		
eGFR (mean ± SD)	63.28 ± 23.52		64.04 ± 24.07		.891		
Medication							
NSAIDs	2	9.5%	25	13.4%	1		
Aspirin	4	19.0%	24	12.8%	.496		
Antiplatelet drugs	7	33.3%	42	22.6%	.284		
Anticoagulants	4	19.0%	40	21.4%	1		
Contrast CT examination							
Extravasation	8	38.1%	15	8.0%	**.002**	0.44 (0.11–1.78)	.251
Colonoscopy							
SRH	8	47.1%*	55	29.4%	.425		
Bleeding source							
Right colon	20	95.2%	55	29.4%	**<.001**	**7.86 (1.6–38.8**)	**.011**
Left colon	1	4.8%	23	12.3%			

Bold values indicates significant differences with <0.05.

BMI = body mass index, BT = blood transfusion, CRP = C-reactive protein, CT = computed tomography, eGFR = estimated glomerular filtration rate, Hb = hemoglobin, IVR = interventional radiology, NSAIDs = non-steroidal anti-inflammatory drug, OR = odds ratio, sBP = systolic blood pressure, SD = standard deviation, SRH = stigmata of recent hemorrhage, WBC = white blood cell.

* Four cases underwent IVR without colonoscopy.

Patients who required TAE (21 cases) were more likely to exhibit a systolic blood pressure of < 80 mm Hg at presentation (*P* = .002), require blood transfusion (*P* = .005), and have a greater blood transfusion volume (*P* = .002) than those who underwent endoscopic hemostasis (66 cases) (Table 2).

**Table 2:** Characteristics of patients who underwent transcatheter arterial embolization and endoscopic hemostasis.

	TAE group		Endoscopic hemostasis group		*P* value
			Clipping (58)		
			Coagulation (2)		
			Ligation (6)		
	n = 21		n = 66		
Age (mean ± SD)	69.33 ± 15.26		69.83 ± 13.35		.886
Male	20	95.2%	50	75.8%	.061
Height (mean ± SD)	165.99 ± 8.2		162.48 ± 9.38		.136
Weight (mean ± SD)	64.2 ± 8.69		64.73 ± 11.83		.853
BMI (mean ± SD)	23.41 ± 3.15		24.39 ± 3.61		.278
Smoking	15	71.4%	32	48.5%	.082
Alcohol drinking	12	57.1%	42	63.6%	.614
Medical history					
Colonic diverticular bleeding	11	52.4%	27	41.5%	.453
Diabetes mellitus	7	33.3%	13	19.7%	.237
Hypertension	13	61.9%	44	66.7%	.793
Dyslipidaemia	4	19.0%	13	19.7%	1
Findings on admission					
Shock (sBP < 80 mm Hg)	8	38.1%	5	7.6%	**.002**
Blood transfusion (BT)	17	81.0%	29	43.9%	**.005**
BT (U)	8 ± 7.32		3.33 ± 5.35		**.002**
Laboratory data					
Hb, g/dL (mean ± SD)	9.51 ± 2.69		10.25 ± 2.62		.267
WBC (mean ± SD)	7738.1 ± 3747.34		7442.42 ± 2804.18		.7
CRP (mean ± SD)	0.37 ± 1.04		0.71 ± 1.58		.355
Creatinine (mean ± SD)	1.23 ± 1.34		1.15 ± 1.25		.82
eGFR (mean ± SD)	63.28 ± 23.52		64.94 ± 24.08		.783
Medication					
NSAIDs	2	9.5%	11	16.7%	.726
Aspirin	4	19.0%	9	13.6%	.505
Antiplatelet drugs	7	33.3%	12	18.5%	.224
Anticoagulants	4	19.0%	12	18.2%	1
Bleeding source					
Right colon	20	95.2%	48	72.7%	.073
Left colon	1	4.8%	17	25.8%	

Bold values indicates significant differences with <0.05.

BMI = body mass index, BT = blood transfusion, CRP = C-reactive protein, eGFR = estimated glomerular filtration rate, Hb = hemoglobin, NSAIDs = non-steroidal anti-inflammatory drug, sBP = systolic blood pressure, SD = standard deviation, TAE = transcatheter arterial embolization, WBC = white blood cell.

*Four cases underwent IVR without colonoscopy.

**Figure 3.**
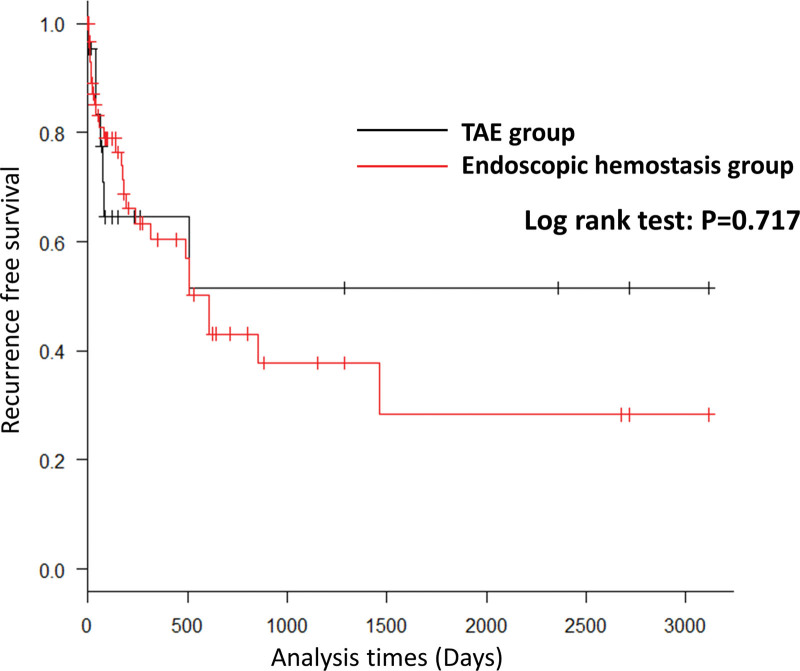
Recurrence-free survival was not significantly different between the TAE and endoscopic hemostasis groups. TAE = transcatheter arterial embolization.

The hemostasis success rate in patients who underwent TAE (85.7%, 18/21) was not significantly different from that in patients who underwent endoscopic hemostasis (84.8%, 56/66) (*P* = 1.0). The early rebleeding rate (*P* = .736), late rebleeding rate (*P* = 1.0) (Table 3), and recurrence-free survival (*P* = .717) (Fig. 3) were not significantly different between the TAE and endoscopic hemostasis groups. In the TAE group, 4 patients (19%) experienced intestinal ischemia and 1 patient (4.8%) experienced colorectal perforation. None of the patients in the endoscopic hemostasis group experienced any complications (*P* < .001) (Table 3).

**Table 3:** Efficacy and safety of transcatheter arterial embolization and endoscopic hemostasis in patients with colonic diverticular bleeding.

	TAE group		Endoscopic hemostasis group		*P* value
	n = 21		n = 66		
Hemostasis success	18	85.70%	56	84.80%	1
Rebleeding					
<30 d	4	19%	10	15.20%	.736
>30 d	4	19%	15	22.70%	1
Complications	5	23.80%	0	0%	<.001
Intestinal ischemia	4	19%	0	0%	
Perforation	1	4.80%	0	0%	

TAE = transcatheter arterial embolization.

**Figure 4.**
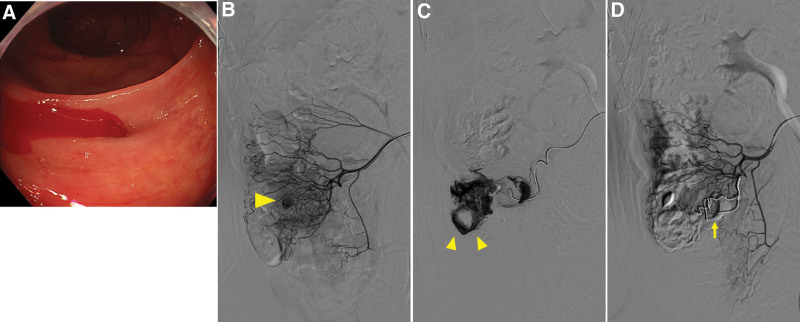
A case of successful transcatheter arterial embolization. Stigmata of recent hemorrhage (SRH) (A) during colonoscopy. Angiography by IVR showed vessels causing aneurysmal dilation during contrast injection (B▲) and revealed vascular leakage (C▲▲). The straight artery and vasa recta were coil-embolized (D↑), and no rebleeding was observed thereafter. IVR = interventional radiology.

**Figure 5.**
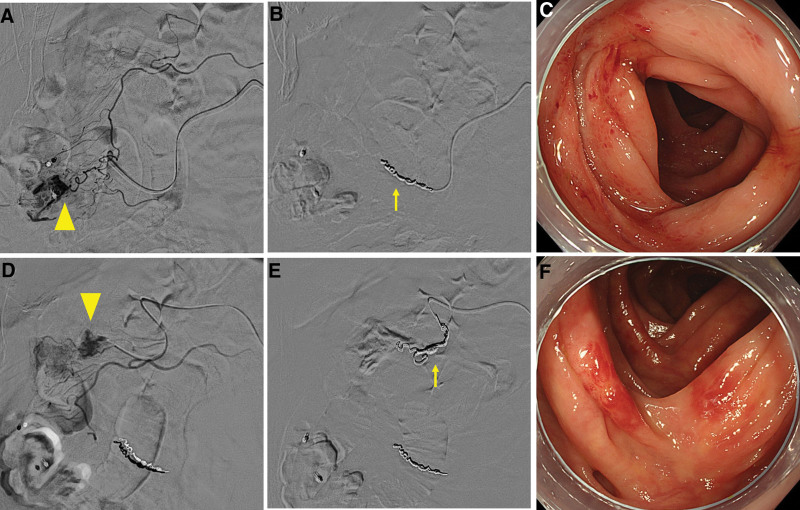
A case of intestinal ischemia after transcatheter arterial embolization. Angiography from the right colonic artery showed vascular leakage (A▲). However, the tortuousness of the straight artery was severe, and the microcatheter did not advance. Hence, a coil was placed in the marginal artery (B↑). Colonoscopy performed at 7 d later revealed occasional shallow erosions (C). Angiography from the middle colic artery showed vascular leakage (D▲), but the catheter was not stable and was coiled over the marginal artery (E↑). Colonoscopy performed at 3 d later showed sporadic erythema and erosion (F).

The details and summary of TAE cases are presented in Tables 4 and 5. In the TAE group, 21 patients (66.7 %) had angiographically confirmed extravasation with a hemostasis success rate of 85.7% (Fig. 4). Hemostasis was not achieved in 3 cases (14.3%), 2 of which required surgery, and one of which died of heart failure. Complications included mild intestinal ischemia in 4 patients (19.0%) (Fig. 5) and perforation requiring surgery in 1 patient (4.8%).

**Table 4:** Details of transcatheter arterial embolization cases.

Age	Sex	Site	CS treatment	CS treatment result	IVR extravasation	TAE	TAE result	Complications	Surgery	Prognosis	(D)	
89	F	Left	Not performed	Not performed	Yes	Coil	Success	No	No	Survival	(7)	*
61	M	Right	Clipping	Failure	No	Coil	Success	Ischaemia	No	Survival	(510)	
78	M	Right	Clipping	Failure	Yes	Coil	Success	No	Yes	Survival	(153)	
60	M	Right	Clipping	Failure	Yes	Coil	Success	No	No	Survival	(3114)	
49	M	Right	Clipping	Failure	No	Coil	Success	No	No	Survival	(1290)	
78	M	Right	Not performed	Not performed	Yes	Coil	Success	No	No	Survival	(232)	*
82	M	Right	Clipping	Failure	Yes	Coil	failure	Ischaemia	Yes	Survival	(260)	
84	M	Right	Clipping	Failure	Yes	Coil	Success	No	No	Survival	(82)	
84	M	Right	Not performed	Not performed	Yes	Coil	Success	No	No	Survival	(76)	*
84	M	Right	Not performed	Not performed	No	Coil	failure	Ischaemia	Yes	Survival	(39)	
61	M	Right	Clipping	Failure	Yes	Coil	Success	No	No	Survival	(2718)	
92	M	Right	Not performed	Not performed	Yes	Coil	failure	No	No	death	(4)	*
69	M	Right	Not performed	Not performed	Yes	Coil	Success	No	No	Survival	(2361)	
55	M	Right	Clipping	Failure	Yes	Coil	Success	No	No	Survival	(38)	
82	M	Right	Not performed	Not performed	No	Coil	Success	No	No	Survival	(66)	
57	M	Right	Not performed	Not performed	No	Coil	Success	Ischaemia	No	Survival	(71)	
77	M	Right	Not performed	Not performed	Yes	Coil	Success	No	No	Survival	(7)	
40	M	Right	Clipping	Failure	No	Coil	Success	No	No	Survival	(8)	
65	M	Right	Not performed	Not performed	Yes	Coil	Success	No	No	Survival	(89)	
43	M	Right	Not performed	Not performed	Yes	Coil	Success	No	No	Survival	(15)	
66	M	Right	Clipping	Failure	No	Coil	Success	Perforation	Yes	Survival	(124)	

CS = colonoscopy, IVR = interventional radiology, TAE = transcatheter arterial embolization.

* IVR without colonoscopy.

**Table 5:** Summary of transcatheter arterial embolization cases.

TAE (n = 21)	IVR extravasation	Success	Complication	Ischaemia	Perforation	Rebleeding	Surgery	Mortality
*n* (%)	14 (66.7)	18 (85.7)	5 (23.8)	4 (19.0)	1* (4.8)	8 (38.1)	4 (19.0)	1** (4.8)

IVR = interventional radiology, TAE = transcatheter arterial embolization.

* Complications requiring surgery.

** Heart failure-related death.

## 4. Discussion

Approximately 70% to 90% of patients with diverticular hemorrhage achieve spontaneous hemostasis.^[7,8]^ Nevertheless, patients with persistent active bleeding may require blood transfusion, arterial embolization, or surgery.^[9,10]^ The mortality rate due to diverticular hemorrhage has been reported to be 0.7% in Japan^[5]^ and 3.9% in Europe, and the United States.^[6]^ An understanding of the risk factors that can be used to predict the severity of diverticular hemorrhage is necessary for the early identification of patients who require further treatment. These risk factors can also be used to determine appropriate treatment methods. However, few studies have reported risk factors that can be used to predict the severity of colonic diverticular bleeding.^[10,22,23]^

Hypotension, tachycardia, syncope, absence of accessory symptoms (such as abdominal pain and diarrhea) associated with bloody stools, history of drug use (including non-steroidal anti-inflammatory drugs, aspirin, and antiplatelet drugs), history of colonic diverticulum or angiodysplasia, history of cardiovascular disease and dementia, and blood test findings (including creatinine, hematocrit, and albumin levels) are predictors of severe bleeding.^[10,22,23]^ This study focused on the risk factors for severe colorectal diverticular bleeding, the most common type of acute lower gastrointestinal bleeding,^[29,30]^ which requires endoscopic hemostasis or TAE.

Based on the results of this study, bleeding from the right-sided diverticula and a systolic blood pressure < 80 mm Hg were risk factors for severe bleeding requiring TAE. Ishii et al reported that the success rate of endoscopic hemostasis was low and IVR was required at a high rate in patients with right-sided diverticular bleeding.^[31]^ Wong et al reported that surgery was required more frequently for right-sided colonic diverticular bleeding than for left-sided colonic diverticular bleeding.^[32]^ Sato et al also showed that a systolic blood pressure of < 90 mm Hg was a risk factor for diverticular bleeding requiring IVR or surgery. Additional risk factors for severe bleeding requiring IVR or surgery in patients with colonic diverticular bleeding include CT extravasation and early rebleeding.^[33]^

Difficulties in endoscopic treatment include cases in which bleeding is stopped but rebleeds repeatedly and cases in which the bleeding site cannot be identified and rebleeds repeatedly. Endoscopic clipping and ligation have recently been reported as endoscopic treatments for colonic diverticular bleeding.^[16]^ Ishii et al showed that ligation was the most effective endoscopic hemostasis technique.^[17]^ Nagata et al reported that EBL was more effective than clipping for the reduction of recurrent colonic diverticular bleeding in the long- and short-term.^[18]^ However, another report indicated that EBL was associated with complications and should be carefully used in select patients.^[16]^

A meta-analysis comparing urgent and elective colonoscopies in patients with acute lower gastrointestinal bleeding showed no significant differences in the rebleeding, mortality, or surgery rates; however, the bleeding source identification rate was significantly higher among patients who underwent urgent colonoscopy.^[34,35]^ The Japanese guidelines for colonic diverticula recommend colonoscopy to be performed within 24 hours after examination to identify the bleeding source and therapeutic intervention.^[3]^ However, Niikura et al reported that colonoscopy performed within 24 hours after admission did not increase the identification rate for SRH or decrease rebleeding, as compared with colonoscopy conducted 24 to 96 hours later, in patients with acute lower gastrointestinal bleeding.^[36]^ Doi et al reported that 96.5% of patients with diverticular bleeding were successfully treated conservatively without early colonoscopy.^[37]^ Recent reports regarding early colonoscopy for diverticular hemorrhage recommend conservative treatment except in severe cases, as the identification rate of SRH was 15% to 42% and the early and late rebleeding rates were not significantly different compared to patients who were treated conservatively.^[36–39]^

In contrast, the identification rate of the bleeding source by angiography is estimated to be 25% to 75%.^[13,40,41]^ In the current study, the identification rate of the bleeding source by angiography was 66.7%, which was considered effective for patients with repeated rebleeding in whom the bleeding source was not identified via colonoscopy. If the bleeding site can be identified, local surgical resection can be performed if endoscopic hemostasis and TAE are unsuccessful.

No significant difference in recurrence-free survival was identified between the IVR and non-IVR groups in this study, which is consistent with the results of a previous study.^[33]^ As pointed out by the authors of the previous study.

The efficacy and safety of TAE for colonic diverticular bleeding were determined by comparing the outcomes of patients who underwent TAE with those of those who underwent endoscopic hemostasis. The TAE group was significantly more likely to have a systolic blood pressure < 80 mm Hg at presentation and to require blood transfusion, with higher transfusion volumes than the endoscopic hemostasis group. There were no significant differences in outcomes, early rebleeding rate, late rebleeding rate, or recurrence-free survival between the groups.

However, in this study, none of the patients who underwent endoscopic hemostasis experienced complications, whereas 23.8% of the patients who underwent TAE did. Ueda et al reported a higher shock index and extravasation on contrast-enhanced CT in patients who underwent TAE than in those who underwent endoscopic hemostasis; however, the outcomes and complications were not compared between the 2 groups.^[42]^

Superselective arterial embolization has a high hemostasis rate (97%) and low intestinal ischemia and rebleeding rates (7% and 15%, respectively).^[24]^ On the other hand, TAE has been reported to be effective for lower gastrointestinal bleeding.^[25–28]^ Complications of TAE include intestinal ischemia and colorectal perforation.^[43]^ In this study, the hemostasis success rate was high in the TAE group, but the complication rate was significantly higher in the TAE group than in the endoscopic hemostasis group. Although arterial embolization should be limited to patients refractory to endoscopic hemostasis because of its cost and complication rate,^[20]^ its high bleeding site identification rate and therapeutic efficacy allow it to be the first-line treatment for patients with massive bleeding and shock. In this study, 75% of the patients (3/4) who were unable to undergo colonoscopy due to shock were successfully treated with TAE.

This study investigated the risk factors for severe colonic diverticular bleeding refractory to endoscopic hemostasis, and evaluated the efficacy and safety of TAE. TAE is an effective treatment for colonic diverticular bleeding but is associated with serious complications. Understanding the risk factors for severe bleeding and the safety of TAE will aid physicians in identifying patients who may benefit from TAE rather than endoscopic hemostasis. This retrospective, single-center study requires further expansion because of the small number of patients included.

In conclusion, patients with colonic diverticular bleeding are more likely to require IVR when the bleeding site is a right-sided colonic diverticulum or when the systolic blood pressure is <80 mm Hg at presentation. Although patients must be monitored carefully for complications, TAE is effective in patients with colonic diverticular hemorrhage that is refractory to endoscopic hemostasis.

## Acknowledgments

We would like to thank Editage (www.editage.cn) for English language editing.

## Author contributions

**Conceptualization:** Tomoe Sano, Toru Ishikawa.

**Formal analysis:** Motoi Azumi.

**Investigation:** Tomoe Sano, Ryo Sato, Ryo Jimbo, Yuji Kobayashi, Toshifumi Sato, Akito Iwanaga, Junji Yokoyama.

**Methodology:** Tomoe Sano, Toru Ishikawa.

**Software:** Tomoe Sano.

**Supervision:** Terasu Honma.

**Writing – original draft:** Tomoe Sano.

**Writing – review & editing:** Tomoe Sano, Toru Ishikawa.
